# Theoretical Prediction of Strengthening in Nanocrystalline Cu with Multi-Element Grain Boundary Segregation Decoration

**DOI:** 10.3390/ma17112504

**Published:** 2024-05-23

**Authors:** Fuli Guo, Chuanying Li, Tao Fu, Xianghe Peng

**Affiliations:** Department of Engineering Mechanics, Chongqing University, Chongqing 400044, China; flguo@cqu.edu.cn (F.G.); cyli@cqu.edu.cn (C.L.)

**Keywords:** high-entropy alloy, grain boundary strengthening, microstructure, mechanical properties

## Abstract

The composition of grain boundaries (GBs) determines their mechanical behavior, which in turn affects the mechanical properties of nanocrystalline materials. Inspired by GB segregation and the concept of high-entropy alloys (HEAs), we investigated, respectively, the mechanical responses of nanocrystalline Cu samples with and without multi-element GBs, as well as the grain size effects, aiming to explore the effects of GB composition decoration on mechanical properties. Our results show that introducing multi-element segregation GBs can significantly improve the mechanical properties of nanocrystalline Cu by effectively inhibiting GB migration and sliding. Additionally, we proposed an improved a theoretical model that can reasonably describe the strengths of the materials with multi-element or single-element segregation GBs. Notably, the introduction of multi-element segregation GBs inhibits both migration and sliding behavior, with migration being more effectively suppressed than sliding. These results present a novel approach for designing high-performance nanometallic materials and offer valuable insights into the role of GB composition decoration in enhancing mechanical properties.

## 1. Introduction

Numerous strategies have been proposed to enhance the mechanical properties of materials, among which grain refinement is one of the most effective approaches. According to the well-known Hall–Petch (HP) relation [1,2], the strength of a polycrystalline material increases as grain size (*d*) decreases, primarily due to the grain boundaries (GBs) hindering dislocation motion [3,4,5,6,7,8]. However, as *d* is decreased to a few or tens of nanometers, the HP relation becomes invalid [9]. For instance, after a critical grain size (*d_cr_*) is reached, typically around 10 to 15 nm for Cu and Ni, the strength begins to decrease, resulting in an inverse Hall–Petch (IHP) relation [9,10]. In this scenario, the GB content increases significantly, and the dominant deformation mechanism shifts from being dislocation-dominated to GB-dominated (GB migration, GB sliding, or grain rotation), leading to softening [11]. In both HP and IHP stages, the mechanical properties of materials are closely linked to the properties of GBs. Therefore, employing some appropriate GB decoration strategy to adjust the behavior of GBs and the interaction between GBs and dislocations can effectively enhance the mechanical properties of polycrystalline materials.

GB decoration strategies can be classified into structural and compositional approaches. Structural strategies usually involve the introduction of low-energy GBs, such as twin boundaries (TBs), to replace general GBs [12]. For instance, Li et al. observed an increase in the strength of Cu and Al-Mg alloy by replacing a portion of general GBs with coherent TBs [13,14]. Compositional strategies, on the other hand, rely on element segregation [15]. Zhou et al. believed that segregation could change the smallest structural hierarchical level, the atomic motifs, which would, in turn, affect the chemical properties of GBs [16]. Peng et al. reported that GB segregation can significantly improve strength, which can further be enhanced by optimizing the solute concentration and GB excess (degree of GB segregation) [17]. Similarly, Hu et al. found that Mo segregation in Ni, induced by annealing, can effectively stabilize high-angle GBs [8]. Multi-element segregation offers a broader range of possibilities for regulating GB performance compared to single-element segregation, potentially leading to more effective improvements. Zhou et al. confirmed, through numerical simulation, that multi-element segregation can reduce the thermodynamic driving force of grain growth [18]. Farkas pointed out that high-entropy GBs, due to multi-element characteristics, possess lower energy and exhibit an optimized structure [19]. Li et al. also demonstrated that the introduction of multiple elements significantly reduces GB energy and atomic density [20]. In addition, Luo et al. used a simplified segregation model to prove that a high-entropy effect can reduce the GB energy of materials as temperature increases. It was also pointed out that high-entropy GBs can stabilize nanocrystalline alloys at high temperatures via thermodynamic and kinetic effects [21]. Among numerous systems, CoCrCuFeNi has been extensively researched [22,23,24], prompting us to choose this system as a representative, with Cu as the matrix, to investigate the impact of introducing multi-element GB on the strength of nanocrystalline materials.

In conventional materials, the HP relation is often used when *d* > *d_cr_*; however, when *d* < *d_cr_*, some theoretical models have been proposed [25,26]. Among them, a simple one without adjusting parameters, which is based on the activation energy (or stress) required to cause deformation via amorphization, was recently suggested by Chandross and Argibay [26] to predict the strength in the IHP stage. Considering the additional strengthening effect of TBs, Xiao et al. [27] and Hu et al. [28] further extended this model to describe the relationship between the strength and characteristic microstructure sizes, such as TB spacing *λ* and grain size *d*, in nanotwinned metals with intracrystalline and intercrystalline TBs. In the former, TBs are introduced into a crystal lattice to enhance the properties of metals, while in the latter, the mechanical properties of materials can be improved significantly by decorating their GB structures with TBs. It should be noted that neither of these strategies alters the chemical composition.

Considering the universality of GB segregation, it is imperative to determine whether multi-element segregation at GBs can effectively improve mechanical properties. If so, it is crucial to establish the corresponding theoretical descriptions to understand this phenomenon. In this work, we present a comprehensive investigation of the influence of multi-element GB segregation on the enhancement of mechanical properties. We firstly confirm the enhancement of mechanical properties resulting from multi-element GB segregation and then conduct a detailed analysis of the underlying strengthening mechanism by analyzing microstructural evolution. Finally, we propose a theoretical model that can reasonably describe the strengthening effects of such GB decorations. The reliability of the theoretical model is also verified in single-element GB segregation materials.

## 2. Method

**Figure 1:**
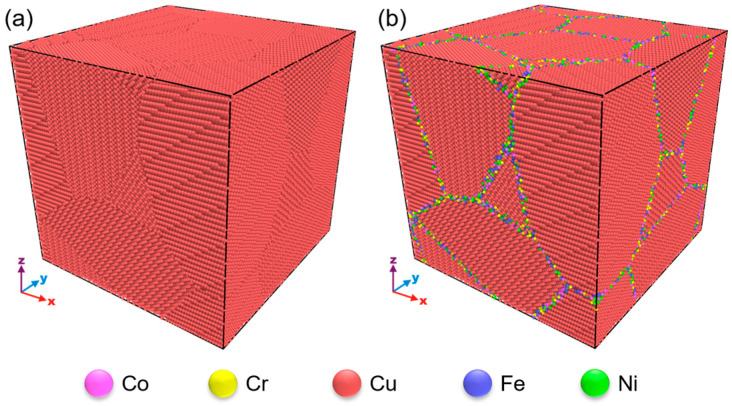
(**a**) Nanopolycrystalline Cu (labeled as pure Cu). (**b**) Multi-element GB segregation Cu (labeled as MES-Cu).

A series of polycrystalline Cu samples with different grain sizes are built using the Voronoi tessellation method in ATOMSK [29]. Subsequently, the centrosymmetry parameter (CSP) [30] of all the atoms is calculated using OVITO [31]. The local lattice disorder of GB atoms is larger than that of grain interior atoms. The CSPs of grain interior atoms approach zero, while those of GB atoms significantly exceed zero. Based on this difference, we identify GB atoms and replace them randomly with an equal proportion of Co, Cr, Fe, Ni, and Cu atoms. As a result, two kinds of nano-polycrystalline Cu samples without (labeled as pure Cu) and with multi-element segregation GB (labeled as MES-Cu) are obtained, as shown in Figure 1a,b, respectively. In each sample, there are eight randomly distributed grains, and the average grain size *d* of the samples ranges from 6.2 nm to 18.6 nm. The difference between pure Cu and MES-Cu is only that the Cu atoms at the GBs of pure Cu are replaced randomly with the same number of equal proportions of Co, Cr, Fe, Ni, and Cu atoms. It should be noted that the purpose of introducing multiple elements only at the GBs is to try not to introduce other elements into the grain interior and avoid introducing intracrystalline solid solution strengthening, so as to focus on the contribution of GB decoration to strength. The embedded atom method potential parameters developed by Farkas and Caro [32] are used to describe the interatomic force. Before deformation, the overlapping atoms are removed to avoid abnormally high local stress induced by improper artificial modeling. Before loading, the samples are heated to 300 K for 20 ps, and then relaxed under NPT ensemble for 20 ps to reach a thermodynamically stable state with zero stress. Finally, a tensile deformation at a strain rate of 1 × 10^8^ s^−1^ is applied along the *x*-direction. During tension, the pressures in the *y*- and *z*-directions are kept at zero. All simulations are performed utilizing the large-scale atomic/molecular massively parallel simulator (LAMMPS) [33]. Periodic boundary conditions are used in all directions.

## 3. Results

### 3.1. Mechanical Response

**Figure 2:**
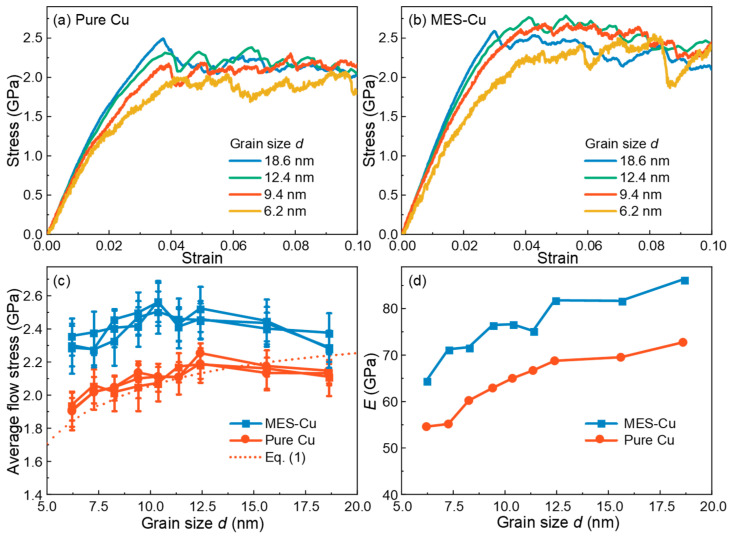
Tensile properties of pure Cu and MES-Cu with various grain size *d* at *T* = 300 K. Stress–strain curves of (**a**) pure Cu and (**b**) MES-Cu; (**c**,**d**) variations of flow stresses (average stress of strain ranged from 0.05~0.1, three sets of samples) and Young’s moduli of pure Cu and MES-Cu with *d*, respectively.

**Figure A1:**
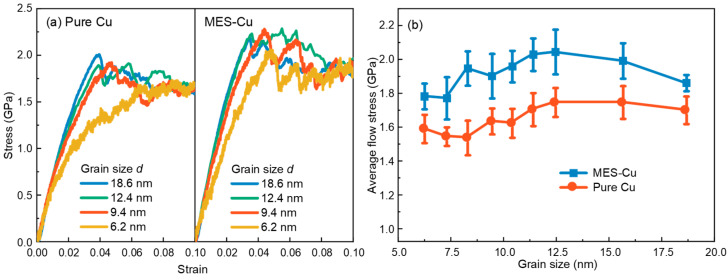
Tensile mechanical response of pure Cu and MES-Cu with different *d* at 500 K. (**a**) Stress–strain curves. (**b**) Variations of average flow stress (strain: 0.05–0.1) of pure Cu and MES-Cu with *d*.

As shown in Figure 2a,b, the stress–strain curves of MES-Cu are significantly higher than those of pure Cu, indicating that multi-element GB segregation can significantly enhance the tensile strength of Cu. Figure 2c shows the variations of the average flow stress of pure Cu and MES-Cu with *d*. To ensure the reliability of the results and guarantee the reproducibility of mechanical property data, we constructed three sets of samples and conducted molecular dynamics (MD) simulations. As can be seen, the differences among the three groups of data are not significant. Both materials exhibit their respective critical grain size, *d_cr_* (the intersection of the HP stage and IHP stage), below which the strength decreases along with the *d* value, indicating a transition from dislocation-dominated to GB-dominated deformation mechanisms [9,34]. Notably, the *d_cr_* of MES-Cu falls between 9.4 nm and 11.35 nm, which is lower than that of pure Cu (11.35 nm < *d_cr_* < 15.6 nm), suggesting that multi-element GB segregation can also delay the IHP relationship of nanocrystalline Cu, similar to that observed in nanocrystalline Cu, with GBs decorated with TBs [28]. Figure 2d shows the variations of the Young’s moduli *E* of pure Cu and MES-Cu with *d*, which increase with the increase in *d*. Generally speaking, the Young’s modulus of GBs is lower than that of grains [35]. With the increase in *d*, the volume fraction of GB decreases, leading to an increase in *E* for both materials. *E* of MES-Cu is higher than that of pure Cu, indicating that *E* of multi-element segregation GBs is higher than that of pure Cu GB. Furthermore, the *E* value obtained in this work is relatively small compared to experimental results because the polycrystalline Cu studied in this work is nanoscale, with small *d* and higher GB volume fraction. GBs with small *E* significantly reduce the *E* of the samples [17,36,37]. Similar multi-element GB segregation strengthening effects are also observed at the temperature of 500 K, as shown in Figure A1 in Appendix A.

### 3.2. Microstructure Evolution: Migration and Sliding of GBs

In the model presented by Chandross and Argibay, based on the activation energy (or stress) required for deformation-induced amorphization, the flow stress of polycrystalline metals with different grain sizes in the IHP stage can be predicted with [26] the following:

(1) $$\tau =\left[\left(L\frac{\rho_{L}}{M}\right)\left(1-\frac{T}{T_{m}}\right)f_{g}-\frac{kT}{b^{3}}\right]+\left(\frac{kT}{{\dot{\varepsilon }}_{\mathrm{tot}}b^{3}}\right){\dot{\varepsilon }}_{\mathrm{GBS}},$$

where *τ* denotes flow stress; *T* the current temperature; *T_m_* the melting point temperature; *L* the heat of fusion; *ρ_L_* the density of the melt at *T_m_*; *M* the atomic mass; ${\dot{\varepsilon }}_{\mathrm{GBS}}$ the strain rate associated with GB sliding [38]; ${\dot{\varepsilon }}_{\mathrm{tot}}$ the total (applied) strain rate; *k* the Boltzmann’s constant; *b* the magnitude of the Burgers vector (equivalent to a metallic atomic diameter) [26]; and *f_g_* = [(*d* − *δ*)/*d*]^3^ the volume fraction of crystalline material in the grains, which incurs an amorphization energy penalty, with *δ* being the thickness of GB that takes the value between 0.5 and 1.0 nm [39,40]. The predicted strength of the samples with different *d* using this model, with the material parameters of Cu [28], are shown in Figure 2c, where they are close to that of the pure Cu sample obtained by MD simulations, but much lower than that of the MES-Cu sample, which suggests that this model cannot consider the contribution of GBs decorated by multi-element segregation. Moreover, in a limit case where the ordered (intragranular) phase in the sample is reduced to an infinitely small size, the material would entirely consist of an amorphous phase, i.e., *f_g_* = 0. Additionally, due to the dynamic simulation (i.e., non-quasi-static), ${\dot{\varepsilon }}_{\mathrm{GBS}}/{\dot{\varepsilon }}_{\mathrm{tot}}≅1$. Substituting these parameters into Equation (1), one can obtain *τ* = 0, which implies that the amorphous material lacks load-bearing capacity, which is obviously unreasonable. It signifies that the model does not adequately consider the contribution of the amorphous phase. Therefore, to accurately predict the strength of fully amorphous samples and the samples with GB segregation, it is imperative to re-consider the deformation mechanism.

**Figure 3:**
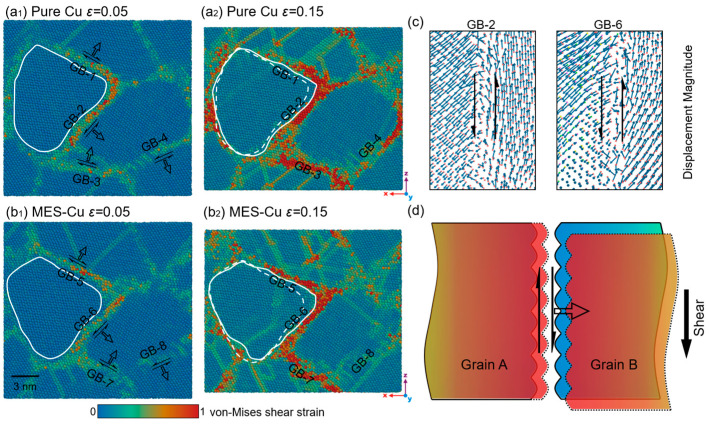
In-plane view of the distribution of atomic von Mises shear strain, (**a**) pure Cu and (**b**) MES-Cu, with which white solid lines representing GB outline at current strain, and dashed lines in (**a_2_**) and (**b_2_**) GB outline at *ε* = 0.05. (**c**) Distributions of displacement vectors at GB-2 and GB-6, as well as the surrounding atoms when *ε* is increased from 0.05 to 0.15, where the arrows represent sliding. (**d**) Schematic illustration of the migration and sliding of GBs, where solid arrows represent sliding and hollow arrows represent migration.

Figure 3a,b show the contour maps of the atomic von Mises shear strain of pure Cu and MES-Cu samples with *d* = 9.4 nm at different applied strains, respectively. At *ε* = 0.05, obvious stacking faults appear in Figure 3(a_1_,b_1_), indicating that the samples are in the post-yield stage. The deformation is more localized at GBs than in the grains, as observed in Figure 3a,b. Consequently, several GBs at the same location in Cu and MES-Cu with a larger atomic strain are selected for analysis and labeled GB-1–GB-4 and GB-5–GB-8, respectively. When *ε* is increased from 0.05 to 0.15, significant migration of these eight GBs occurs, with their sliding and migration directions marked using black hollow arrows and solid arrows, respectively, as shown in Figure 3(a_1_,b_1_).

To observe the migration behavior more clearly, GB-1 and GB-2 in the Cu sample, and GB-5 and GB-6 in the MES-Cu sample, are selected for further analysis. The GB outline at the current strain is represented with white solid lines, while the dashed lines in Figure 3(a_2_,b_2_) indicate the GB outlines at *ε* = 0.05. Figure 3(a_2_,b_2_) show clearly that GB-1, GB-2, GB-5, and GB-6 migrate significantly. Figure 3c shows the distributions of the displacement vectors at GB-2 and GB-6, as well as the surrounding atoms when *ε* is increased from 0.05 to 0.15. The analysis of the displacements of the intergranular atoms reveals a distinct slip of the GBs, while the displacement directions of atoms in different grains are roughly the same. Figure 3d schematically illustrates the migration and sliding behavior of GBs, with blue and red areas indicating the positions of the grains before and after deformation.

The analysis conducted above demonstrates notable GB migration and sliding during the plastic deformation of both pure Cu and MES-Cu. Further quantification of the effects of GB migration and sliding should be crucial for evaluating the strengthening mechanisms of the materials.

## 4. Discussion

### 4.1. Derivation of Theoretical Model

In the study by Chandross and Argibay [26], Equation (1) is derived from

(2) $${\dot{\varepsilon }}_{\mathrm{GBS}}={\dot{\varepsilon }}_{\mathrm{tot}}\exp\left(-\frac{\Delta F-\tau V^{*}}{kT}\right),$$

where V* is the activation volume and Δ*F* is the activation energy,

(3) $$\Delta F=\left(L\frac{\rho_{L}}{M}\right)\left(1-\frac{T}{T_{m}}\right)f_{g}V^{*},$$

in which *ρ_L_* is used as an approximation of the density of the amorphous phase. It is well known that the basic mechanism of GB migration is a transfer of a group of atoms from the lattice of one grain to the disordered state at the GB and a subsequent transfer of a group of atoms from the disordered boundary layer to the crystalline lattice of another grain [41]. This migration requires overcoming an energy barrier, which can be regarded as the energy difference between the GB atoms and the atoms in the grain. In our work, all the GBs involved are high-angle GBs. According to the Mott’s “island” model [42], high-angle GBs are composed of many “islands” where the atoms are arranged neatly and separated by regions with a relatively chaotic atomic configuration. Amorphous materials, as revealed by X-ray and electron diffraction measurements [43,44,45,46,47,48,49,50,51,52,53,54], are composed of efficiently packed clusters and regions with relatively chaotic atoms between clusters [55]. From a structural perspective, the two share similarities, and the high-angle GB in this work can be approximated to the amorphous phase. Consequently, the activation energy of GB migration can be regarded as the energy difference between the crystalline and amorphous phases [42,56,57,58,59]. By analyzing Equation (3), we can see that Δ*F* is related to the heat of fusion *L*, representing the energy difference between the crystalline and amorphous phases [42]. Hence, *L*(*ρ*_L_/*M*) represents the energy required to transform per-unit volume of the lattice structure into an amorphous phase. By multiplying [*L*(*ρ*_L_/*M*)]*f_g_* with the activation volume of *b*^3^ [60] of single atomic motions, one can obtain the activation energy for the transformation from the crystal phase to the amorphous phase. Additionally, when the temperature reaches the melting point *T_m_*, the material undergoes complete melting, with no transition from the ordered phase to the amorphous phase, aligning with Equation (3), where Δ*F* = 0 when *T* = *T_m_*. Consequently, it can be inferred that the amorphization model [26] describes the mutual transformation between an ordered phase and an amorphous phase (GB phase), i.e., GB migration rather than GB sliding.

The microstructural analysis indicates that both pure Cu and MES-Cu exhibit notable GB migration and GB sliding during plastic deformation, which dominates the softening in the IHP stage. Therefore, it is necessary to consider the softening from the two mechanisms when predicting strength. It should be noted that although both mechanisms belong to GB behavior, the sources of their contributions are different: grain interior and GB. Their influence on the overall strength is closely related to their respective volume fractions. Therefore, we use the rule of mixtures based on volume fraction to predict the overall stress. Additionally, although there may be coupling between the two mechanisms, we simplify the treatment in this work and ignore the coupling for the time being. Inspired by the work by Xiao and Deng [27], we express the stress in nanocrystalline metals by considering the softening induced by both GB migration and GB sliding simultaneously as follows:

(4) $$\tau =\tau_{\mathrm{GB}-M}f_{g}+\tau_{\mathrm{GB}-S}f_{gb},$$

where *τ*, *τ*_GB-M_, and *τ*_GB-S_ are the stresses for the whole material, GB migration and GB sliding, respectively; *f_g_* and *f_gb_* are the volume fractions of grain interior and GB, respectively, with *f_g_* + *f_gb_* = 1.

It should be noted that the GB sliding mentioned by Chandross and Argibay [26] is, in fact, GB migration. Therefore, we use similar expressions to describe GB migration, i.e., the corresponding strain rate ${\dot{\varepsilon }}_{\mathrm{GB}-M}$ can be expressed as follows:

(5) $${\dot{\varepsilon }}_{\mathrm{GB}-M}={\dot{\varepsilon }}_{\mathrm{tot}}\exp\left(-\frac{\Delta F-\tau_{\mathrm{GB}-M}V^{*}}{kT}\right),$$

with

(6) $$\Delta F=\left(L\frac{\rho_{L}}{M}\right)\left(1-\frac{T}{T_{m}}\right)V^{*}.$$

The difference between Equations (6) and (3) lies in the absence of *f*_g_, which is due to the fact that the contribution of GB has already been taken into account in Equation (4).

Substituting Equation (6) into (5), one can solve

(7) $$\tau_{\mathrm{GB}-M}=\left(L\frac{\rho_{L}}{M}\right)\left(1-\frac{T}{T_{m}}\right)+\frac{kT}{V^{*}}\ln\frac{{\dot{\varepsilon }}_{\mathrm{GB}-M}}{{\dot{\varepsilon }}_{\mathrm{tot}}}.$$

Furthermore, it is necessary to reevaluate the effect of GB sliding on strength, particularly in materials that are strengthened by GB decoration such as segregation. Similar to GB migration, the transition of atoms at GBs into a sliding state requires energy to destroy the original structure. The strain rate ${\dot{\varepsilon }}_{\mathrm{GB}-S}$ associated with GB sliding can be expressed as follows:

(8) $${\dot{\varepsilon }}_{\mathrm{GB}-S}={\dot{\varepsilon }}_{\mathrm{tot}}\exp\left(-\frac{\Delta F_{\mathrm{GB}-S}-\tau_{\mathrm{GB}-S}V_{\mathrm{GB}-S}^{*}}{kT}\right).$$

Inspired by Equation (6), we describe the activation energy Δ*F*_GB−S_ with

(9) $$\Delta F_{\mathrm{GB}-S}=\left(\Delta E\frac{\rho }{M_{\mathrm{GB}}}\right)\left(1-\frac{T_{\mathrm{GB}}}{T_{\mathrm{GB}-m}}\right)V_{\mathrm{GB}-S}^{*},$$

where Δ*E* is the energy difference between the initial equilibrium state and the sliding state; *ρ* the density of GB atoms in the sliding state; *M*_GB_ the atomic mass of GBs; *T*_GB_ the current temperature; and *T*_GB-m_ the melting temperature of GB (assuming that the strength of GBs varies linearly with temperature). Combining Equation (8) with Equation (9), one obtains

(10) $$\tau_{\mathrm{GB}-S}=\left(\Delta E\frac{\rho }{M_{\mathrm{GB}}}\right)\left(1-\frac{T_{\mathrm{GB}}}{T_{\mathrm{GB}-m}}\right)+\frac{kT}{V_{\mathrm{GB}-S}^{*}}\ln\frac{{\dot{\varepsilon }}_{\mathrm{GB}-S}}{{\dot{\varepsilon }}_{\mathrm{tot}}}.$$

Substituting Equations (7) and (10) into Equation (4) yields

(11) $$\tau =\left(L\frac{\rho_{L}}{M}\right)\left(1-\frac{T}{T_{m}}\right)f_{g}+\left(\Delta E\frac{\rho }{M_{\mathrm{GB}}}\right)\left(1-\frac{T_{\mathrm{GB}}}{T_{\mathrm{GB}-m}}\right)f_{gb}+\frac{kT}{V^{*}}\ln\frac{{\dot{\varepsilon }}_{\mathrm{GB}-M}}{{\dot{\varepsilon }}_{\mathrm{tot}}}f_{g}+\frac{kT}{V_{\mathrm{GB}-S}^{*}}\ln\frac{{\dot{\varepsilon }}_{\mathrm{GB}-S}}{{\dot{\varepsilon }}_{\mathrm{tot}}}f_{gb}.$$

In MD simulations, it is usually assumed that the ratio ${\dot{\varepsilon }}_{\mathrm{GB}-S}/{\dot{\varepsilon }}_{\mathrm{tot}}≅1$ and ${\dot{\varepsilon }}_{\mathrm{GB}-M}/{\dot{\varepsilon }}_{\mathrm{tot}}≅1$ [26,27], which makes the last two terms on the right-hand side (RHS) of Equation (11) vanish, so Equation (11) is reduced to the following:

(12) $$\tau =\left(L\frac{\rho_{L}}{M}\right)\left(1-\frac{T}{T_{m}}\right)f_{g}+\left(\Delta E\frac{\rho }{M_{\mathrm{GB}}}\right)\left(1-\frac{T_{\mathrm{GB}}}{T_{\mathrm{GB}-m}}\right)f_{gb}.$$

The theoretical model is derived based on the physical principle that any change in the internal structure of a material requires overcoming some energy barrier. Stable structures are typically situated at the saddle points of the energy curve, which has a lower energy level. In this work, we observe two primary microstructure evolution behaviors during deformation, GB migration and GB sliding, which involve the transition of the material’s structure from one stable configuration to the other under deformation. Essentially, there exists an energy barrier in this process, and plastic deformations can only occur after overcoming this barrier, as described by the thermal activation theory [26,27,28,42,61,62].

### 4.2. Validation of Theoretical Model

The material parameters in the first term on the RHS of Equation (12) can be determined by direct calculation. For pure Cu, the value of each parameter of Cu in the migration term can be calculated directly, as listed in Table 1, with which the value of the migration term can be calculated as 1.426 GPa. However, for MES-Cu samples, GBs are the edges of grains, and small amounts of other elements are observed in the Cu crystal phase during deformation. In this case, we calculate the proportion of other elements in the crystals of different grain sizes during the IHP stage and find that the values do not differ much, so the average is taken, and the value is 0.05. Subsequently, we calculate the material parameters of the Cu crystal containing other elements, substitute them into [*L*(*ρ_L_*/*M*)](1 − *T*/*T_m_*), and find that the value of this term is increased to 1.59 GPa.

Determining the material parameters in the second term on the RHS of Equation (12), such as Δ*E*, *T*_GB-m_, etc., is another challenge. Here, an alternative method is proposed. Based on the previous discussion in Section 4.1, one can see that high-angle GBs and the amorphous phase share structural similarities. Furthermore, from the perspective of the deformation process, GB sliding represents an activation process that transforms the initial state of the “island” model into a flow state [42]. Similarly, the shear deformation of the amorphous phase is an activation process that transforms the amorphous phase from an efficiently packed solid state of atomic clusters into a randomly packed, liquidlike configuration [55]. This perspective is supported by studies by Argon [63] and Spaepen [64], further emphasizing the similarities in the deformation process. Consequently, we associate GB sliding with the shear deformation of amorphous materials. When *f_g_* = 0, indicating the absence of the crystalline phase, it can be assumed that all intracrystalline phases in the sample have been transformed into an amorphous phase. In this scenario, *f_gb_* = 1 − *f_g_* = 1, and Equation (12) describes the strength of the amorphous phase. Therefore, by measuring the strength of the amorphous phase, we can obtain the value of *τ*_GB-S_ = *τ*_amor_ = [Δ*E*(*ρ*/*M*_GB_)] (1 − *T*_GB_/*T*_GB-m_), i.e., GB sliding can be approximately equivalent to the amorphous flow. Then, Equation (12) turns into the following:

(13) $$\tau =\left(L\frac{\rho_{L}}{M}\right)\left(1-\frac{T}{T_{m}}\right)f_{g}+\tau_{\mathrm{amor}}\left(1-f_{g}\right).$$

According to the Mises equivalence rule $\sigma =\sqrt{3}\tau$, the strength under uniaxial tension can be expressed as follows:

(14) $$\sigma =\sqrt{3}\left[\left(L\frac{\rho_{L}}{M}\right)\left(1-\frac{T}{T_{m}}\right)f_{g}+\tau_{\mathrm{amor}}\left(1-f_{g}\right)\right].$$

**Figure 4:**
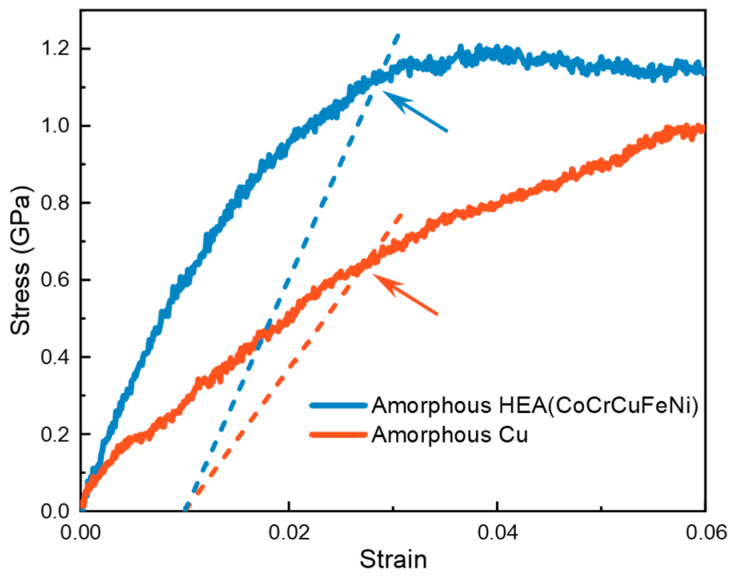
Stress–strain curves of amorphous Cu and amorphous CoCrCuFeNi at 300 K, with arrows indicating 1% offset yield stress.

To determine the yield stress of the amorphous phase, the following steps are taken. Initially, the polycrystalline sample is heated until it completely melts, and then the melt undergoes relaxation to achieve a thermodynamically stable state, followed by rapid solidification through extremely quick cooling to form the amorphous phase. Subsequently, the stress–strain curves of amorphous CoCrCuFeNi and amorphous Cu at a strain rate of 1 × 10^8^ s^−1^ at 300 K are obtained using MD simulation, as shown in Figure 4. It is evident that the amorphous high-entropy alloy (HEA) exhibits a distinct yield point, while the amorphous Cu does not. Furthermore, the stress of the amorphous Cu continues to increase with the increase in strain, attributed to significant crystallization during deformation. In engineering, when the yield point of a metallic material is unobvious, the stress at which the residual strain reaches 0.2% (0.2% offset stress) is commonly considered as the offset yield strength. However, in the case of small grain sizes, the 0.2% offset criterion is not suitable to define the yield strength [65], as it fails to reflect the onset of deformation in nanostructures [66]. Extensive research suggests that, for MD simulation, the point that reflects the onset of plastic deformation typically falls between 0.7% and 1.2% [66,67,68,69]. After careful analysis, a value of 1% is adopted. Upon application, it is observed that, for amorphous CoCrCuFeNi, the 1% offset stress aligns closely with the yield stress value obtained directly, validating the choice of 1%. Therefore, the 1% offset stress is employed as the yield strength ($\sqrt{3}\tau_{\mathrm{amor}}$). For amorphous Cu, this value is 0.64 GPa, while for amorphous CoCrCuFeNi, it is 1.12 GPa.

**Figure 5:**
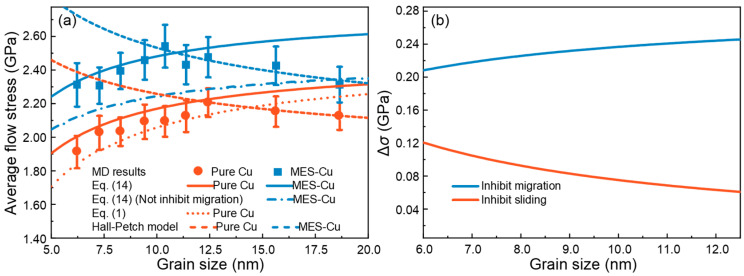
(**a**) Theoretical prediction of the average flow stress of pure Cu and MES-Cu at 300 K. (**b**) Contributions to strength (Δ*σ*) from inhibiting GB migration and GB sliding.

The GB width *δ* of pure Cu and MES-Cu sample are taken as 0.58 nm and 0.59 nm, respectively. Hence, all parameters in Equation (14) have been obtained. It can be seen in Figure 5a that the improved theoretical model [Equation (14)] used to predict the average flow stress in the IHP stage can describe the strength of both pure Cu and MES-Cu better than the amorphization model [Equation (1)]. It should be noted that the flow stress shown in Figure 5a is the average of the flow stresses of a set of three samples [Figure 2c]. Furthermore, during the derivation of the theoretical model, we temporarily ignore the coupling of the two behaviors, which may introduce some error. However, it can be found in the results that such errors are within an acceptable range.

### 4.3. Effect of Migration and Sliding on Strength

Compared to pure Cu, the average flow stress in the IHP stage of MES-Cu stands out due to the introduction of multi-element segregation GBs. It can be seen in Equation (14) that the strength of the material is influenced by GB migration and GB sliding. In MES-Cu, the contribution of GB migration and sliding terms is larger than that of pure Cu, as evidenced by the following results: 1.59 GPa > 1.426 GPa and 1.12 GPa > 0.64 GPa. Thus, it can be inferred that the introduction of multi-element segregation GBs can not only inhibit GB sliding but also GB migration. To further analyze whether inhibiting sliding or inhibiting migration contributes more to the improvement of material strength, a curve without considering the inhibitory effect of multi-element segregation on GB migration is plotted with a dash-dotted line in Figure 5a, which is obtained using the material parameters for Cu in the first term on the RHS of Equation (14) and the yield stress of the amorphous HEA in the second term on the RHS of Equation (14). In other words, this curve only takes into account the inhibitory effect on sliding and ignores the inhibitory effect on migration.

**Figure A2:**
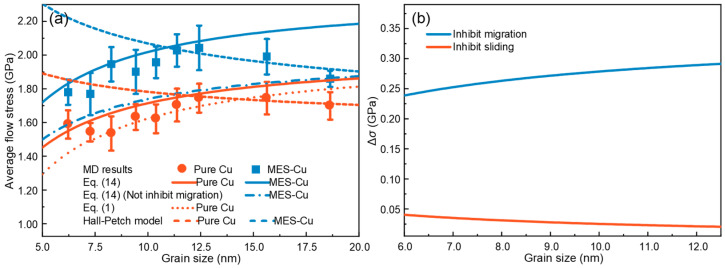
(**a**) Theoretical prediction of average flow stress of pure Cu and MES-Cu at 500 K. (**b**) Contributions to strength (Δ*σ*) from inhibiting GB migration and inhibiting GB sliding.

By comparing the dash-dotted line with the two solid lines that represent the strength of pure Cu and MES-Cu, it can be inferred that the enhancement of the strength resulting from inhibiting GB migration is larger than that from inhibiting GB sliding, as shown in Figure 5a. To provide a more intuitive representation of the contributions by these two factors, the contributions to strength (Δ*σ*), resulting from inhibiting GB migration and inhibiting GB sliding, are shown, respectively, in Figure 5b for the grain size ranging from 6.0 nm to 12.5 nm, corresponding to the IHP stage. It is evident that the contribution from inhibiting GB migration consistently outweighs the contribution from inhibiting GB sliding. Moreover, it is worth noting that with the increase in grain size, the Δ*σ* resulting from inhibiting GB sliding decreases, while the Δ*σ* resulting from inhibiting GB migration increases. This is because the effects of migration and sliding on the overall strength are related to the grain interior and GB, respectively, and the GB fraction decreases with the increase in grain size. Taking into account the effect of temperature, the theoretical descriptions of the two materials at *T* = 500 K are also carried out [Figure A2a in Appendix B], where Equation (14) can also describe the strength of the IHP stage of the two materials well. It can be observed in Figure A2a,b in Appendix B that, at 500 K, the introduction of multi-element segregation GBs has a larger suppression effect on GB migration compared to GB sliding. This further confirms the feasibility of the theoretical model. 

Upon comparing the contributions of the two deformation behaviors at different temperatures, it can be seen that, at a low temperature of 300 K, the values of the migration term and the sliding term are relatively high. However, at a high temperature of 500 K, these values decrease. Specifically, the GB migration term decreases from 1.426 GPa to 1.166 GPa for pure Cu, and from 1.59 GPa to 1.366 GPa for MES-Cu. Similarly, the GB sliding term decreases from 0.64 GPa to 0.44 GPa for pure Cu, and from 1.12 GPa to 0.59 GPa for MES-Cu. This indicates that with an increase in temperature, the migration and sliding of GBs become easier. Furthermore, to assess the dominance of the two mechanisms, we conduct a comparative analysis of Figure 5a and Figure A2a and calculate the ratio of the sliding term to the migration term for the two materials at different temperatures (i.e., *τ*_amor_/{[*L*(*ρ_L_*/*M*)](1 − *T*/*T_m_*)}). It can be found that, as the temperature is decreased from 500 K to 300 K, for pure Cu, the ratio increases from 0.218 to 0.259; while for MES-Cu, the ratio increases from 0.249 to 0.407. This shows that with a decrease in temperature, the dominant degree of GB sliding increases, and correspondingly, the dominant degree of GB migration decreases.

In addition, it can also be seen that the flow stress in the MES-Cu samples is larger than that in pure Cu in the HP stage, as shown in Figure 5a. Fitting the points in the HP stage in Figure 5a with *σ* = *σ*_0_ + *kd*^−1/2^, the result shows that the *σ*_0_ values of pure Cu and MES-Cu are 1.77 GPa and 1.81 GPa, respectively, where *σ*_0_ is the resistance to dislocation motion in the grain interior [70]. The slightly higher value observed for MES-Cu can be attributed to the presence of a small amount of other elements within its grain interior. This result is consistent with the research of Zhang et al., and the values are roughly the same [71]. In addition, the value of *k* obtained from the fitting of MES-Cu is 2.28 GPa nm^1/2^, which is significantly higher than the *k* value of pure Cu, which is 1.54 GPa nm^1/2^. The same situation also appears in the case of 500 K [Figure A2a]. This shows that multi-element segregation GB can improve performance not only in the IHP stage, but also in the HP stage.

**Figure 6:**
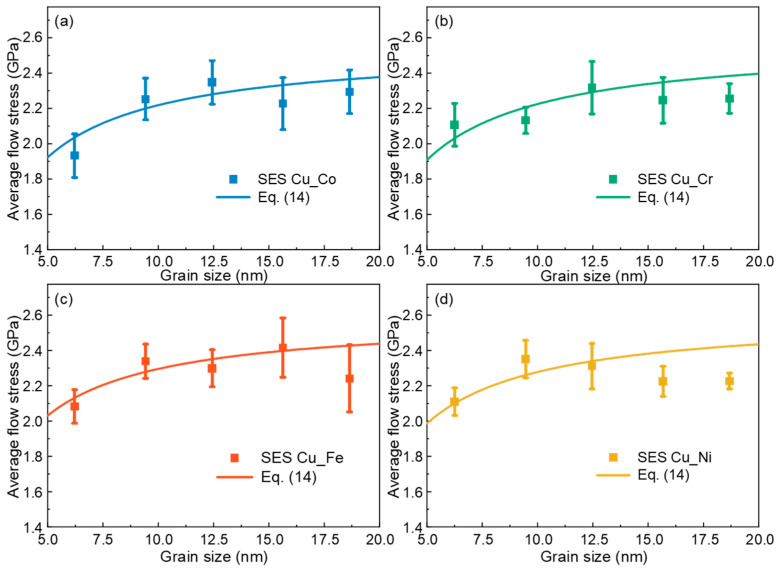
Theoretical predictions of the average flow stress of nanocrystalline Cu with GBs containing 20 at.% randomly distributed atoms of (**a**) Co, (**b**) Cr, (**c**) Fe and (**d**) Ni, respectively.

Furthermore, we also verify the applicability of the new theoretical model in the single-element segregation samples. Four polycrystalline samples with GBs containing, respectively, 20 at.% randomly distributed Co, Cr, Fe and Ni atoms (denoted as SES Cu_Co, SES Cu_Cr, SES Cu_Fe and SES Cu_Ni, respectively) are constructed. The mechanical responses of the samples under uniaxial tension at a strain rate of 1 × 10^8^ s^−1^ at 300 K are simulated using MD and shown in Figure 6. The results demonstrate that the theoretical model can describe the average flow stress of the four single-element segregation samples well.

**Figure 7:**
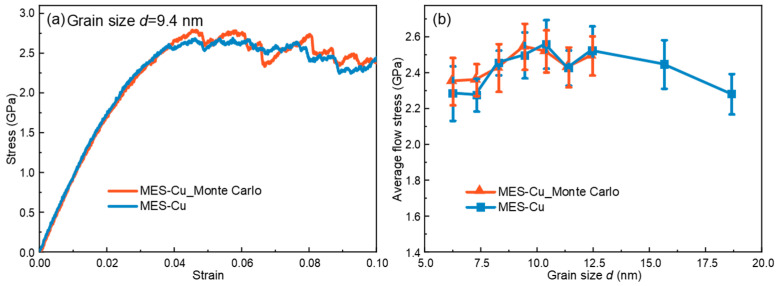
(**a**) Stress–strain curves of the MES-Cu sample of *d* = 9.4 nm with and without MC simulation. (**b**) Comparison of average flow stress-grain size curves for MES-Cu with and without MC simulation.

The element distribution may affect the results. Therefore, we utilize Monte Carlo (MC) simulations to construct additional samples. After energy minimization and relaxation, we conduct mechanical testing simulations. Figure 7a shows the stress–strain curves of MES-Cu with *d* = 9.4 nm with and without MC simulation, respectively, which shows minimal difference. Furthermore, the average flow stresses of the MES-Cu of the samples with different grain sizes in the IHP stage are presented in Figure 7b, showing that the average flow stress curve obtained by MC simulation is slightly higher than that without MC simulation, but the impact is not significant. Therefore, we can conclude that performing MC simulations does not have any notable impact on the results in this study.

## 5. Conclusions

In summary, we investigated, respectively, the mechanical responses of nanocrystalline Cu samples without and with multi-element segregation GB (pure Cu and MES-Cu), as well as the grain size effect. Both pure Cu and MES-Cu exhibit HP and IHP effects, but the critical grain size of MES-Cu is smaller than that of pure Cu. The flow stress of MES-Cu is larger than that of pure Cu in both the IHP and HP stages, indicating that multi-element GB is an effective strategy to improve the mechanical properties of nanocrystalline metals. The microstructural analysis showed that GB migration and GB sliding dominate the mechanical properties of the materials in the IHP stage. Finally, an improved model that takes into account the effect of GB sliding and GB migration was proposed, which can better describe the strength of nanomaterials with multi-element segregation GBs. It is observed in our analysis that the introduction of multi-element segregation GBs can inhibit both GB migration and sliding behaviors, and the inhibition effect on GB migration is stronger than that on GB sliding. In addition, the reliability of the theoretical model is also verified in single-element segregation materials.

The application scope of the improved model is suitable not only for pure metals and binary alloys, but also for GB decoration materials (such as segregation [17,72,73], amorphous intergranular films [74,75]), and even an amorphous phase. In our work, we temporarily ignored the coupling effect between GB migration and GB sliding, and the results indicate that the error is within an acceptable range. In our subsequent research, we will be committed to developing a more comprehensive theory by employing bicrystal samples to investigate the coupling effect between these two mechanisms in deeper level. Additionally, the strength of the amorphous materials in this study is obtained using MD simulations, and future studies may explore the direct calculations of amorphous strength using theoretical models that describe it [55]. This work is of great significance for understanding the mechanical properties and deformation mechanism of multi-element GB segregation Cu. Additionally, it provides a new idea and method for developing and optimizing high-performance nanocrystalline metals.

## Figures and Tables

**Table 1:** Material parameter.

	*L* (kJ/mol)	*ρ_L_* (g/cm^3^)	*M* (g/mol)	*T_m_* (K)
Pure Cu	15.64	7.38	63.546	1397
MES-Cu	16.39	7.334	62.111	1699

## Data Availability

The raw data supporting the conclusions of this article will be made available by the authors on request.

## Appendix A. Tensile Mechanical Properties of Pure Cu and MES-Cu with Different Grain Size at 500 K

Considering that the strength of nanocrystalline metals decreases with an increase in *T* [76,77,78], we take *T* into account and randomly increase the *T* of samples to explore the strengthening effect of multi-element GB segregation on the mechanical properties of nanocrystalline Cu after the *T* increase, so as to make the study more rigorous. Figure A1 shows the stress–strain curves of pure Cu and MES-Cu with different grain sizes at 500 K, where it can be seen that the flow stress of the MES-Cu samples is higher than that of pure Cu. Figure A1 shows the variations of the flow stress of pure Cu and MES-Cu with grain size at 500 K. Compared with the results at 300 K (Figure 2c), the critical grain size between the HP and IHP stages shifts forward.

## Appendix B. Applicability of the Theoretical Model in Pure Cu and MES-Cu at 500 K

To consider the influence of temperature *T*, we calculate the strength of pure Cu and MES-Cu at 500 K. It is evident in Figure A2a that the improved theoretical model effectively describes the strength of both materials. In addition, further analysis reveals that the introduction of multi-element segregation GB effectively inhibits both GB migration and GB sliding. From Figure A2b, it can be definitively concluded that inhibiting GB migration plays a more significant role in enhancing strength compared to inhibiting GB sliding.
